# Co-occurrence of CT-based radiological sarcopenia and frailty are related to impaired survival in surgical oncology

**DOI:** 10.1093/bjr/tqaf023

**Published:** 2025-02-08

**Authors:** Linda B M Weerink, Barbara L van Leeuwen, Thomas C Kwee, Claudine J C Lamoth, Barbara C van Munster, Geertruida H de Bock

**Affiliations:** Department of Epidemiology, University Medical Center Groningen, 9700 RB Groningen, The Netherlands; Department of Radiology, Regional Hospital Queen Beatrix, 7101 BN Winterswijk, The Netherlands; Department of Surgery, University Medical Center Groningen, 9700 RB Groningen, The Netherlands; Department of Radiology, University Medical Center Groningen, 9700 RB Groningen, The Netherlands; Department of Human Movement Sciences, University Medical Center Groningen, 9700 RB Groningen, The Netherlands; Department of Internal Medicine, University Medical Center Groningen, 9700 RB Groningen, The Netherlands; Department of Epidemiology, University Medical Center Groningen, 9700 RB Groningen, The Netherlands

**Keywords:** radiological sarcopenia, CT imaging, frailty, oncology, survival

## Abstract

**Objectives:**

The objective of this study was to investigate the association of radiological sarcopenia and frailty with postoperative outcomes in adult patients undergoing oncological surgery.

**Methods:**

Data were derived from the PICNIC study, consisting of two consecutive series of patients undergoing surgical cancer treatment. Radiological sarcopenia was assessed based on CT imaging. The presence of low muscle mass and/or low muscle density was determined based on lowest quartile gender specific cut-off values. Frailty was defined by a score of ≥4 on the Groningen frailty index. Postoperative overall survival was analysed with Kaplan-Meier curves and Logrank testing. Multivariable Cox regression analyses adjusted for age and gender were performed to calculate adjusted hazard ratios (HR).

**Results:**

A total of 372 patients were included. Median age was 69 (28-86) years, 77 patients (23.5%) were frail and radiological sarcopenia was present in 134 patients (41.0%). Combined radiological sarcopenia and frailty was present in 35 patients (10.7%). One-year (65.6% versus 87.0%) and three-year survival (31.4% versus 66.8%) were significantly worse in patients with combined radiological sarcopenia and frailty. The combined presence of radiological sarcopenia and frailty was associated with significantly decreased overall survival (HR_adjusted_: 2.06, 95% CI: 1.39-3.05, *P* < .001).

**Conclusion:**

Co-occurrence of radiological sarcopenia and frailty is strongly related to impaired survival in surgical cancer patients.

**Advances in knowledge:**

The combined presence of radiological sarcopenia and frailty is associated with decreased postoperative survival, strongly exceeding the effects of both risk factors separately. The use of radiological sarcopenia in addition to frailty screening can further optimize preoperative risk stratification.

## Introduction

Surgery is an important treatment modality for most solid malignancies. Although the treatment is often very effective, it may lead to adverse outcomes including morbidity and even mortality. The risk of developing postoperative complications and negative sequelae is greater in frail patients,^1–3^ and the presence of sarcopenia has emerged as an additional risk factor.^4–6^

Frailty is a multifaceted geriatric syndrome defined by decline in one or multiple domains including the physical, cognitive, and social domain which is present in 5%-17% of the community dwelling older persons.^7^ Frail older patients often present with the presence of multiple chronic conditions, weakness, and fatigue. The presence of frailty is associated with marked vulnerability and a higher risk on adverse outcomes.^3^^,^^8^^,^^9^ Sarcopenia, on the other hand, is solely a muscle disorder, in the physical domain, characterized by muscle wasting, ie, decline of muscle mass and muscle quality, and loss of muscle function and strength. Both conditions are frequently present in older patients and low grip strength, slow gait speed, and weight loss are common in both conditions. Frailty is assessed using one of the frailty screening tools, including all domains of the frailty syndrome.^3^ The presence of sarcopenia can be assessed using clinical tests and/or the estimation of the skeletal muscle status.^9^ Radiological imaging, eg, CT-based imaging, is widely used to estimate the muscle status, and it can be used to detect low muscle mass and low muscle density as signs of radiological sarcopenia.^6^^,^^10^

Low skeletal muscle mass is a hallmark feature of sarcopenia.^9^ Research shows that muscle mass can be improved through preoperative physical training programs, which might result in a lower complication rate.^11^^,^^12^ This makes screening for sarcopenia worthwhile, as detection may lead to participation in physical training programs with a potential for the improvement of postoperative outcomes. However, it is not clear how often sarcopenia and frailty co-exist and whether the presence of sarcopenia enhances the negative effect of frailty on postoperative outcomes. Therefore, the role of (radiological) sarcopenia measurements to frailty screening in preoperative risk stratification remains unclear. The aim of this study was to evaluate the effects of radiological sarcopenia and frailty on postoperative outcomes in adult patients undergoing surgical treatment for a solid malignancy.

## Methods

**Figure 1.**
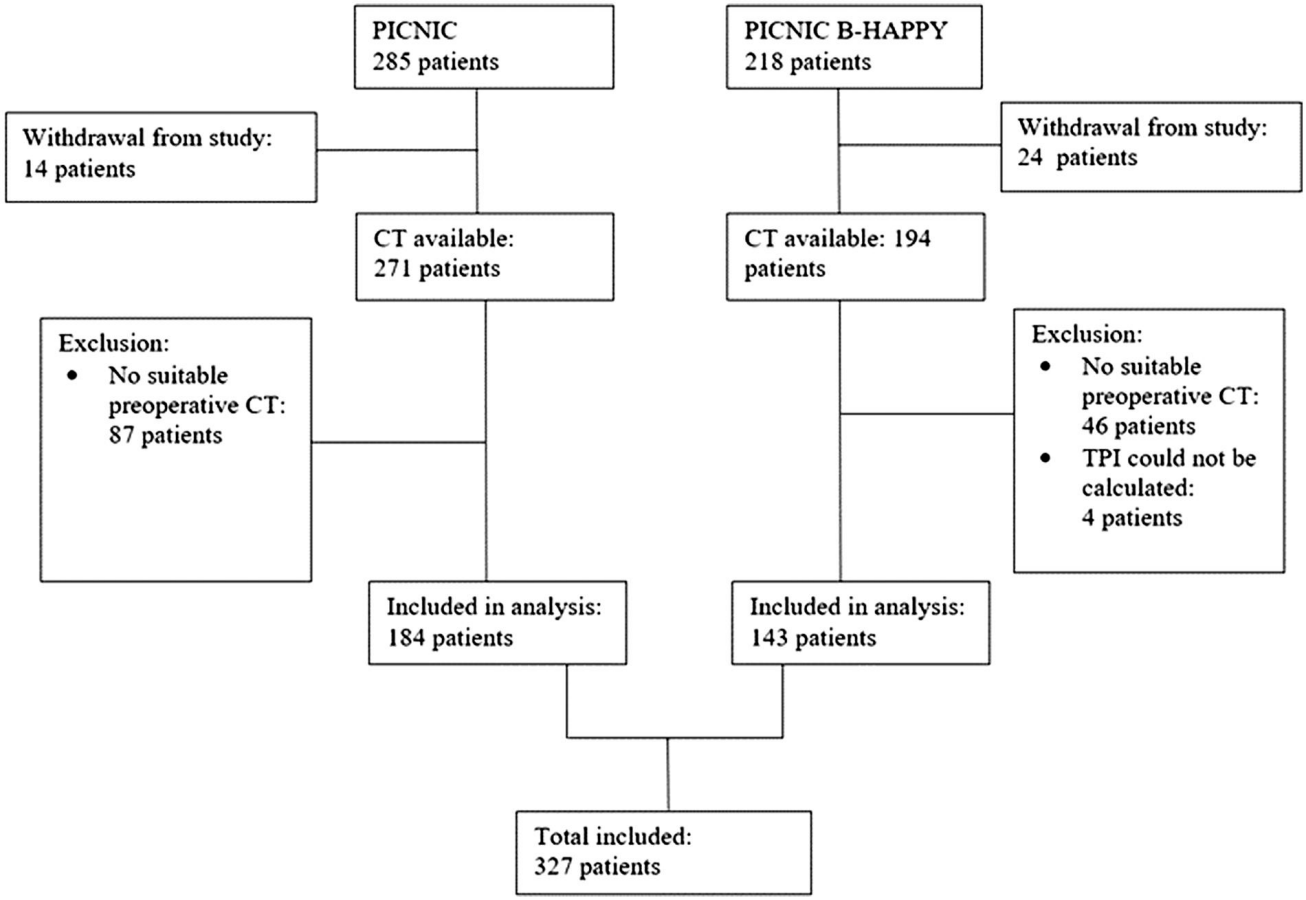
Inclusion scheme PICNIC and PICNIC B-HAPPY study patients.

This study is part of the prospective observational PICNIC studies including patients aged 65 years and older in the PICNIC-1 study cohort and patients of all ages in the PICNIC-BHAPPY cohort (Figure 1).^13–16^ The PICNIC study was conducted at the University Medical Center Groningen (Groningen, the Netherlands) with the inclusion period ranging from 2010 to 2017. Adult patients of any age with a solid malignancy in the gynaecological tract, digestive tract, soft tissues, and other organs deemed eligible for surgical removal were enrolled in this study. Exclusion criteria were: any physical condition potentially hampering compliance with the study protocol, such as a severe visual or auditory impairment; insufficient understanding of the Dutch language; pre-existing cognitive impairment (ie, preoperative diagnosis of dementia); and a recent history of stroke with severe loss of cognition and function. Patients did not participate in a prehabilitation program prior to surgery. Patients were included after written informed consent. The research protocol was approved by the Medical Ethical Committee of this hospital and was registered under trial number NL45602.042.14.

All patients from the PICNIC study cohorts with suitable preoperative CT imaging were included in the analysis (Figure 1). The two main outcomes were: severe postoperative complications and mortality. Postoperative complications occurring within 30 days after surgery, including 30-day mortality, were graded using the Clavien-Dindo scale (CD).^17^ The CD scale is used to grade postoperative surgical complications based on the therapy or interventions needed. Grade 1 is minor deviations of the postoperative course not requiring further treatment. Grade 2 complications require pharmacological treatment, grade 3 complications require (invasive) interventions; grade 4 complications require critical care management and grade 5 complications are those complications leading to the patient’s death.^18^ Complications with a CD score 1-2 were considered minor complications, ie, urinary tract infection treated medically, and complications graded CD score 3-4-5 were considered major complications, ie, complications treated with surgical interventions or admission to the intensive care unit. To assess whether a patient was still alive, data were retrieved from the Dutch basic registration of persons and addresses (June 2022).

Potentially predictive variables were radiological sarcopenia and frailty. Radiological sarcopenia was defined as the presence of low muscle mass and/or low muscle quality.

**Figure 2.**
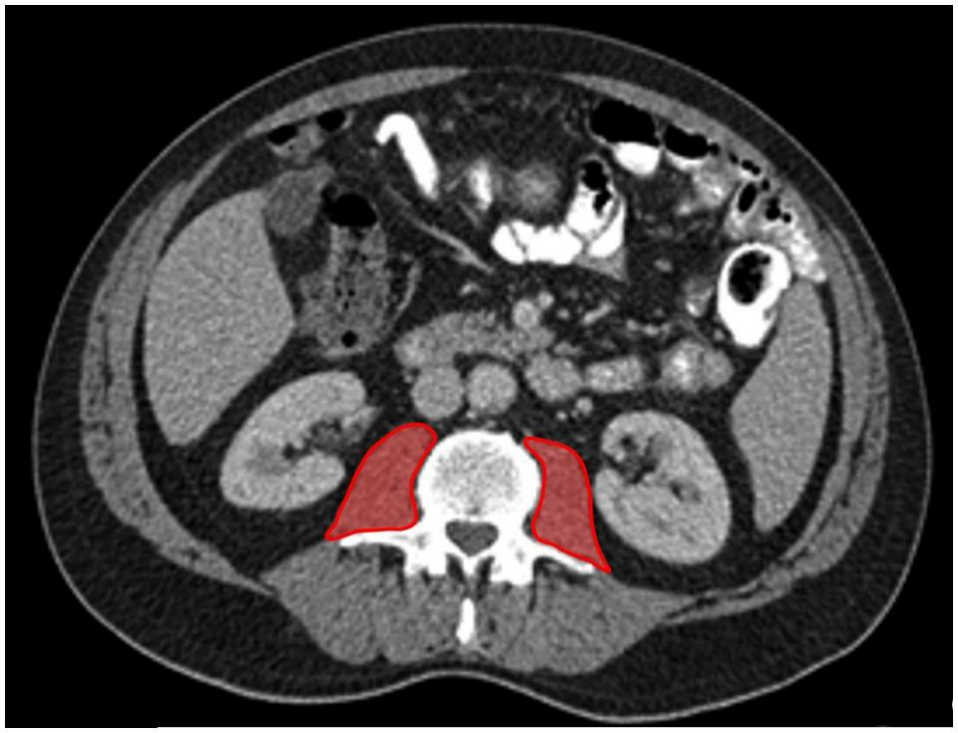
Total Psoas area.

The presence of radiological sarcopenia was determined using CT imaging. Scans were performed 60-70 s after intravenous administration of iodinated contrast medium. Muscle mass was evaluated using the total psoas index (TPI, cm^2^/m^2^). To calculate the TPI, the total psoas area (TPA, cm^2^) was first determined by outlining both psoas muscles and calculation of the total surface area (Figure 2). The TPI was then calculated: TPI (cm^2^/m^2^) = TPA (cm^2^)/patient height^2^ (m^2^).^19^ Muscle quality was assessed by measuring the radiodensity (Hounsfield Units) of the psoas muscles (total psoas radiodensity, TPRD), which was automatically calculated within the outlined area. Measurements were performed on the most cranial image that clearly showed both transverse processes of the L3 vertebra, with the thinnest available slice thickness, typically 1 mm.^10^ Outlining of the psoas muscles was performed manually by a radiologist (LW) and trained researcher (MM) using Aquarius Intuition software.^20^^,^^21^ Cut-off values for low muscle mass and muscle quality were determined based on the gender specific 25-percentile, based on methods described in literature.^22^

Frailty was assessed using the Groningen frailty index (GFI), which was performed by trained nurses as part of the preoperative workup. The GFI is a 15-question questionnaire with scores ranging from 0 to 15 that assesses the cognitive, physical, social, and psychological domains.^23^ Patients with a score ≥4 were considered frail.

Age, gender, BMI, and the presence of comorbidities were considered relevant confounders. A BMI > 25 was considered overweight. Tumour location was registered by subgroup. Comorbidities were classified using the Charlson comorbidity index (CCI); a score ≥4 was considered a high burden of comorbidity. The American Society of Anaesthesiologist (ASA) score was used to gain insight in the physical status of the patients; patients were stratified in ASA I-II versus ASA III-V.^24^

### Statistical analysis

The intra- and inter-observer agreement for determining the TPA and the correlation between radiological sarcopenia and frailty was calculated with Spearmans’ correlation coefficient (*ρ*). The association between radiological sarcopenia, frailty, and severe postoperative complications was evaluated with uni- and multivariable logistic regression analyses. Odds ratios (ORs) with 95% confidence intervals (95% CI) were calculated. The association between sarcopenia, frailty, and overall postoperative survival is presented with Kaplan-Meier curves and analysed using Logrank testing. Additional univariate and multivariable Cox regression analyses were performed, and hazard ratios (HR) with 95% CIs were calculated. All analyses were performed for radiological sarcopenia and frailty separately and combined. A *P*-value <.05 was considered statistically significant. All statistical analyses were performed with SPSS version 23 (SPSS Inc., Chicago, IL, United States).

## Results

### Patient characteristics

A total of 503 patients were enrolled in the PICNIC study cohorts, and 327 patients were included in this analysis (Figure 1). The median age was 69 years (range 26-89 years), and 173 (52.9%) patients were males (Table 1).

**Table 1.** Patient and disease characteristics.

Variable	Patients (*n* = 327)
Age (median, range)	69.0 (26-89 years)
Male gender	173 (52.9%)
BMI >25	211 (64.5%)
CCI ≥4^a^	169 (51.7%)
ASA score^b^	
ASA 1-2	234 (71.6%)
ASA 3-4-5	91 (27.8%)
Frailty^c^	77 (23.5%)
Tumour location	
Colorectal	95 (29.1%)
Upper gastrointestinal tract	71 (21.7%)
Gynaecological	55 (16.8%)
Pancreas and hepatobiliary	49 (15.0%)
Other	56 (17.2%)
Tumour stage	
Stage 0	7 (2.1%)
Stage I	63 (19.3%)
Stage II	53 (16.2%)
Stage III	83 (25.4%)
Stage IV	85 (26.0%)
Sarcopenia	
Radiological sarcopenia^d^	134 (41.0%)
Radiological sarcopenia and frailty	35 (10.7%)

a Charlson comorbidity index.

b American Society of Anesthesiologists score.

c Groningen frailty index score ≥4.

d The presence of low muscle mass and/or low muscle quality.

In males, the gender specific cut-offs were TPI <5.9 cm^2^/m^2^ for low muscle mass and TPRD <41.5 HU for low muscle density. In females, the cut-off values were TPI <4.7 cm^2^/m^2^ and <42.8 HU, respectively. Radiological sarcopenia was present in 134 (41.0%). A total of 77 (23.5%) patients had a GFI ≥4 and were considered frail. The combination of radiological sarcopenia and frailty was present in 35 patients (10.7%).

The majority of patients was classified with an ASA 1-2 score (71.6%). The CCI score was ≥4 in 51.7%. Colorectal carcinomas were the most common subgroup of tumour locations (29.1%).

### Radiological sarcopenia and frailty

**Figure 3.**
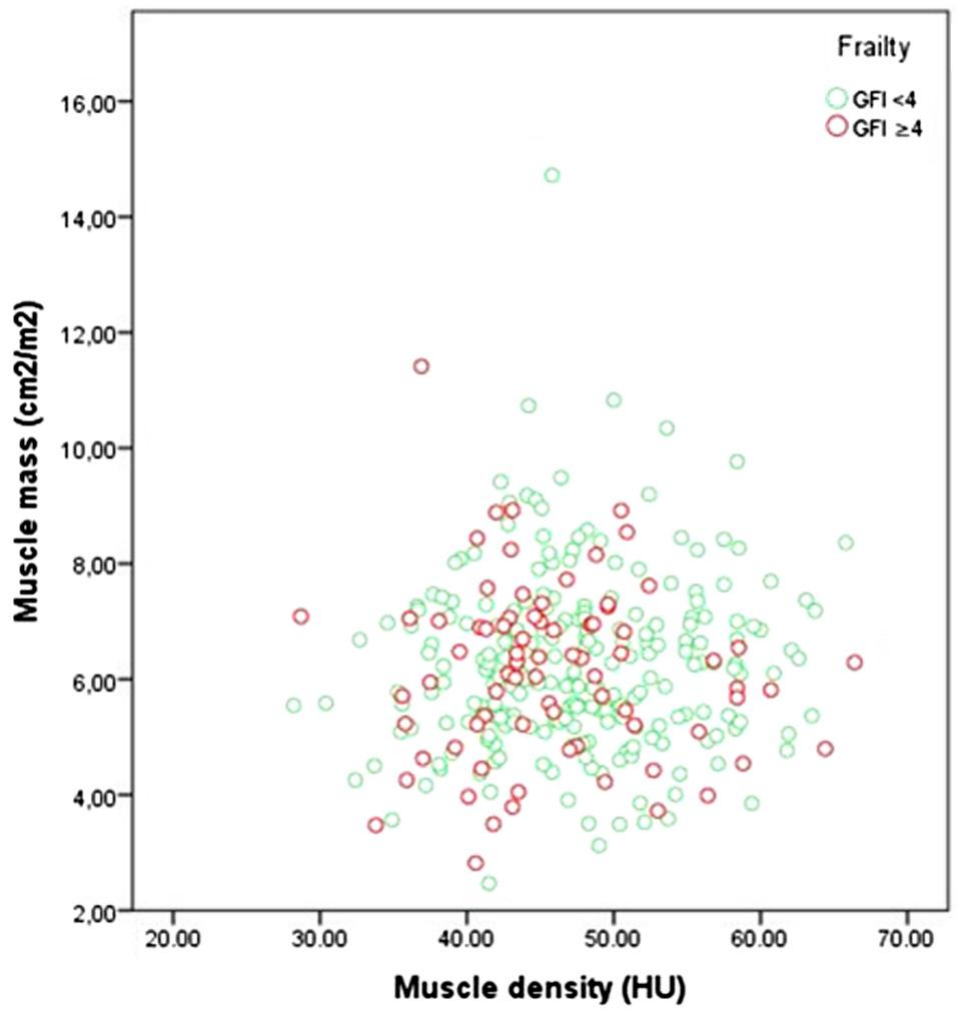
Correlation between radiological sarcopenia and frailty.

The intra-observer agreement for determining the psoas area was *ρ* 0.96 and the interobserver agreement *ρ* 0.91. The correlation between radiological sarcopenia and frailty was *ρ* 0.48 (Figure 3). In most cases, the GFI was assessed at the same day the CT imaging was performed (52.3%), with a maximum range of 12 days between the assessments. Patients with combined radiological sarcopenia and frailty were more often aged 70 years and over (60.0% versus 41.4%, *P*: .04; Table 2). There were no differences between gender and tumour location in patients with and without the combined presence of radiological sarcopenia and frailty (Table 2).

**Table 2.** Differences between patients with and without both radiological sarcopenia and frailty.

Variables	No radiological sarcopenia and frailty (*n* = 292)	Combined radiological sarcopenia and frailty (*n* = 35)	*P*-value^a^
Age > 70 years	121 (41.4%)	21 (60.0%)	.04
Male gender	158 (54.1%)	15 (42.9%)	.21
Most common tumor location			.44
Colorectal	86 (29.5%)	9 (25.7%)	
Upper gastrointestinal tract	64 (21.9%)	7 (20.0%)	
Pancreas and hepatobiliary	43 (14.7%)	6 (17.1%)	

a Chi^2^ test.

### Postoperative outcomes

**Figure 4.**
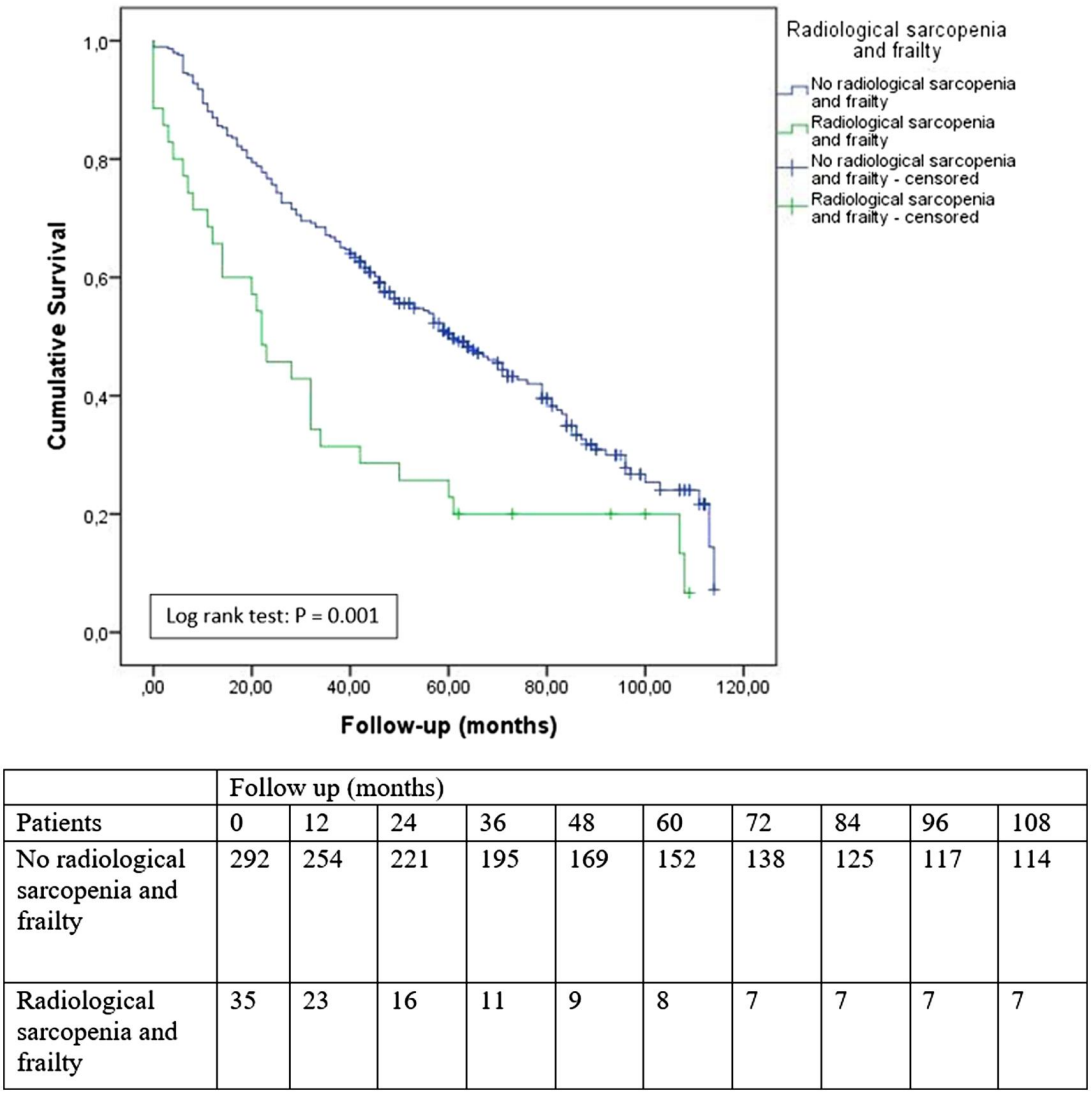
Survival of patients with radiological sarcopenia and frailty.

The mean duration of follow-up was 50 months (range 0-114 months). Postoperative complications occurred in 151 (46.2%) of the patients, of which 43 (13.1%) severe postoperative complications, and a 30-day mortality of 2.1% (Table 3). The one-year survival was 84.7% and the three-year mortality was 63.0% (Table 3). In patients with radiological sarcopenia and frailty (*N* = 35) the 30-day mortality was 11.4%. The one-year survival was 65.6% and three-year mortality was 31.4% (*P* < .001) (Table 3, Figure 4).

**Table 3.** Postoperative outcomes.

Variables	Overall (*n* = 327)	Combined radiological sarcopenia and frailty (*n* = 35)	No combined radiological sarcopenia and frailty (*n* = 292)
Postoperative complications			
All complications	151 (46.2%)	17 (48.6%)	134 (45.9%)
C-D^a^ 1-2	108 (33.0%)	11 (31.4%)	97 (33.2%)
C-D 3-4-5	43 (13.1%)	6 (17.1%)	37 (12.7%)
Postoperative mortality^b^			
30-day mortality	7 (2.1%)	4 (11.4%)	3 (1.0%)
One-year survival	50 (84.7%)	12 (65.6%)	38 (87.0%)
Three-year survival	121 (63.0%)	24 (31.4%)	97 (66.8%)

a Clavien-Dindo classification.

b Percentages derived from Kaplan-Meier curves.

Radiological sarcopenia solely was not associated with the development of severe postoperative complications (OR_unadjusted_: 1.04, 95% CI: 0.54-2.00, *P*: .91) or decreased survival (HR_unadjusted_: 1.19, 95% CI: 0.91-1.56, *P*: .21). The presence of frailty was a predictor for the development of severe postoperative complications (OR_adjusted_: 2.13, 95% CI: 1.03-4.41, *P*: .04) and worse postoperative survival (HR_adjusted_: 1.38, 95% CI: 1.00-1.89, *P*: .05) (Table 4). The combined presence of radiological sarcopenia and frailty was not associated with the development of severe postoperative complications, but it was strongly associated with impaired postoperative survival (HR_adjusted_: 2.06, 95% CI 1.39-3.05, *P*: <.001) (Table 4).

**Table 4.** Radiologic sarcopenia and frailty as predictors for postoperative outcomes.

Severe postoperative complications				
	Unadjusted estimates^a^		Adjusted estimates	
Variables	OR (95% CI)	*P*-value	aOR (95% CI)	*P*-value
Radiological sarcopenia	1.04 (0.54-2.00)	.91	1.29 (0.62-2.72)^b^	.50
Frailty	2.16 (1.05-4.42)	.04	2.13 (1.03-4.41)^c^	.04
Radiological sarcopenia and frailty	1.36 (0.53-3.53)	.52	1.33 (0.51-3.49)^c^	.56

Postoperative survival				
	Unadjusted estimates^d^		Adjusted estimates	
Variables	HR (95% CI)	*P*-value	aHR (95% CI)	*P*-value
Radiological sarcopenia	1.19 (0.91-1.56)	.21	1.13 (0.86-1.50)^b^	.38
Frailty	1.35 (0.99-1.84)	.06	1.38 (1.00-1.89)^c^	.05
Radiological sarcopenia and frailty	2.03 (1.38-3.00)	<.001	2.06 (1.39-3.05)^c^	<.001

a Logistic regression analysis.

b Adjusted for age, gender, GFI score.

c Adjusted for age, gender.

d Cox regression analysis.

## Discussion

This study showed that in this heterogeneous cohort of 327 oncological patients of all ages, the co-occurrence of radiological sarcopenia and frailty was 10% (*N* = 35). In patients with this co-occurrence, postoperative survival was significantly impaired. One-year survival was 66% in patients with radiological sarcopenia and frailty, versus 87% in patients without the presence of both radiological sarcopenia and frailty. Three-year survival was also significantly worse: 31% versus 67%. Patients with radiological sarcopenia and frailty were more often aged over 70 years (60%) compared to patients without radiological sarcopenia and frailty.

In our population, the number of patients with low psoas muscle mass was relatively low, 24% versus 33%-43% as reported in the literature.^4^ The number of patients with low muscle quality in our study (26%) was also at the lower end of the reported range (11%-85%).^6^ In addition, the co-occurrence of radiological sarcopenia and frailty was relatively rare. These findings may be explained by the relatively good physical condition of the included patients. Only patients deemed fit enough for curative surgical treatment who consented to participate in the extra pre- and post-operative assessments as part of the study protocol were included. This resulted in a relatively fit study group, mostly ASA class I and II patients, with relatively few comorbidities and good performance status. Furthermore, the correlation between radiological sarcopenia and frailty was low to moderate. This emphasizes the fact that (radiological) sarcopenia and frailty are distinct conditions and should not be used interchangeably.^9^

The adverse effects of frailty and radiological sarcopenia on postoperative outcomes are well documented individually.^3^^,^^4^^,^^25^^,^^26^ Surprisingly, in our study, radiological sarcopenia alone was not related to adverse postoperative outcomes. This may have been caused by the combined use of low muscle mass or muscle density to determine radiological sarcopenia. This resulted in a relatively large group of patients with radiological sarcopenia (41.0%), which lowers the discriminative value between those patients with and without an increased risk of postoperative morbidity and mortality. CT imaging is widely used to determine the skeletal muscle status in order to detect (radiological) sarcopenia. In this study, we used the psoas muscle mass over the total skeletal muscle mass since it has a greater predictive value for development of adverse postoperative outcome.^4^ Even though consensus about the cut-off values for the total skeletal muscle index is emerging, discussion about the exact measurement method remains.^27^ A commonly used method to determine cut-off values for low psoas mass and/or density is the use of the lowest gender specific quartiles, but agreement about the cut-off values is lacking.^27^^,^^28^ Additional research is needed to determine those cut-off values to optimize the detection of radiological sarcopenia and to further improve its use in preoperative risk stratification. The association between frailty and adverse postoperative outcomes observed in our study is in line with literature.^26^

Our finding that the combined presence of radiological sarcopenia and frailty is associated with worse survival is supported by findings presented by Buettner et al.^29^ Their study presented a nomogram which showed that the combination of skeletal muscle measurements and clinical factors leads to a better identification of those patients with the highest one-year mortality risk.^29^ In our study, we found that the differences between mortality ratios between patients groups with and without combined radiological sarcopenia and frailty increased over time. This indicates that the combined presence of radiological sarcopenia and frailty signals increased vulnerability, and its effects are associated with both a decreased short- and long-term survival.

An important strength of this study is the preoperative assessment of radiological sarcopenia and frailty at, nearly, the same time, providing a broader estimation of the preoperative condition of the patients. Furthermore, the use of both radiological sarcopenia and frailty separately and combined in multivariate analysis gives insights in their respective roles as preoperative risk factors. A limitation of this study is the fact that this study cohort consisted of a selected and relatively fit group of patients. However, any type of (oncological) surgery requires a certain level of physical fitness. Since similar selection processes are present in routine oncological care the findings of this study are deemed applicable in the general population.

Both frailty and sarcopenia are more common in the older population, and their prevalence increases with age.^30^ Frailty has been reported in 4.1% of adults aged 50-64 years and 17% in people aged 65 years and over.^30^ In people aged over 90 years, the prevalence of frailty rises to 65%.^30^ Furthermore, sarcopenia has been reported to be present in 5%-13% of the people aged 60-70 years and up to 50% in people aged 80 years and over.^31^ Given these data in the general population, the higher prevalence of combined radiological sarcopenia and frailty in older patients compared to the younger patients in our study population is in accordance with these findings.

The higher prevalence of combined radiological sarcopenia and frailty in older patients and its effects on postoperative outcomes warrants a greater vigilance in the older population. In current guidelines for surgery in older patients, a geriatric risk assessment is advised in patients aged 70 years and over.^32^ Since the presence of combined radiological sarcopenia and frailty is associated with a 2-fold increased postoperative mortality risk compared to frailty alone, it can be argued that in addition to the geriatric risk assessment radiological sarcopenia should be determined in patients who are considered vulnerable. This information can further optimize the process of preoperative shared decision making. It can be an additional tool in selecting those patients in which the perioperative risk of extensive surgery exceeds the potential gains. For instance, the identification of patients who might benefit more from less extensive, eg, palliative, procedures instead of extensive surgery with curative intent, will result in more custom-made care. It remains to be determined whether frailty and (radiological) sarcopenia are modifiable prior to surgery, especially in the older, ie, aged 70 years and over, population. Prehabilitation programs with physical exercise training prior to surgery show promising results.^12^^,^^33^ However, it remains unclear if these programs are applicable to a frail older population and whether they also decrease the vulnerability of the involved patients on the longer term.

In summary, the co-occurrence of radiological sarcopenia and frailty is associated with a significantly impaired postoperative survival. The combination of radiological sarcopenia and frailty occurs more often in the older population, underlining the increased vulnerability of this patient group.

## Supplementary Material

tqaf023_Supplementary_Data
