# Supplementary material for: Co-occurrence of CT-based radiological sarcopenia and frailty are related to impaired survival in surgical oncology
Source: Br J Radiol. 2025 Feb 8;98(1168):607–13. doi: 10.1093/bjr/tqaf023 (PMC11919072; doi:10.1093/bjr/tqaf023)
Supplement: tqaf023_Supplementary_Data [file tqaf023_supplementary_data.zip › tqaf023_Supplementary_Data/Supplementary table 1 RS frailty.docx]

**Supplementary table 1: Postoperative outcomes in patients with only radiological sarcopenia or only frailty**

| **Variables** | | **Only radiological sarcopenia without frailty (n=89)** | **Only frailty without radiological sarcopenia (n=42)** |
| --- | --- | --- | --- |
| **Postoperative complications** | | | |
| All complications | | 31 (34.8%) | 22 (59.5%) |
|  | C-D^a^ 1-2 | 23 (25.8%) | 13 (31%) |
|  | C-D 3-4-5 | 8 (9.0%) | 9 (21.4%) |
| **Postoperative mortality^b^** | | | |
| 30-day mortality | | 2 (2.2%) | 1 (2.4%) |
| 1-year survival | | 12 (86.5%) | 7 (83.3%) |
| 3-year survival | | 28 (68.5%) | 16 (61.9%) |
| a: Clavien-Dindo classification  b: percentages derived from Kaplan-Meier curves | | | |
